# Parent-Child Relationship of Pedometer-Assessed Physical Activity and Proxy-Reported Screen Time in Czech Families with Preschoolers

**DOI:** 10.3390/ijerph13070740

**Published:** 2016-07-21

**Authors:** Erik Sigmund, Petr Badura, Jana Vokacova, Dagmar Sigmundová

**Affiliations:** Institute of Active Lifestyle, Faculty of Physical Culture, Palacký University Olomouc, Tr. Miru 117, Olomouc 77111, Czech Republic; petr.badura@upol.cz (P.B.); jana.vokacova@upol.cz (J.V.); dagmar.sigmundova@upol.cz (D.S.)

**Keywords:** step count, screen time, Yamax pedometer, fathers, mothers, weekdays, weekends

## Abstract

This study focuses on determining the relationship between parents’ step count (SC) and screen time (ST) and children’s SC and ST on weekdays and at weekends. The participants (278 parents aged 30–45 and their 194 children aged 4–7) were recruited from 10 randomly selected Czech kindergartens. The participants recorded SC and ST duration over a week-long monitoring (≥8 h/day) during September–October 2014 and April–May 2015. The associations between parents’ SC and ST and children’s SC and ST were estimated using general linear regression for weekdays and weekends. Each 2500 SC increase in mothers’/fathers’ daily SC at weekdays (weekends) was associated with an extra 1143/903 (928/753) daily SC in children. Each 60 min of ST increase in mothers’/fathers’ ST at weekdays (weekends) was associated with an extra 7.6/7.6 (16.8/13.0) min of child daily ST. An increase of 2500 mothers’ daily SC was associated with reduction of 2.5 (7.5) min of ST in children at weekdays (weekends). This study reveals a significant relationship between parent-child SC/day, parent-child ST/day, and mothers’ ST and children’s SC at weekends. Weekend days seem to provide a suitable space for the promotion of joint physical activity in parents and their pre-schoolers.

## 1. Introduction

Preschool age is a crucial period for the formation of healthy lifestyle habits [1,2]. However, the worldwide incidence of overweight/obesity among pre-schoolers in 2010 reached 6.7% and researchers predicted that there would be 60 million (9.1%) overweight or obese pre-schoolers worldwide by 2020, with estimated prevalence of 14.1% in developed countries [3]. Moreover, obesity acquired in preschool age has a significant tendency to persist into adulthood [4,5,6]. Although childhood obesity has arisen due to a complex array of interactions among multiple behavioral, biological, and environmental factors [7], excessive screen time (ST) and low level of physical activity (PA) are often discussed as crucial factors [8,9,10]. Thus, active lifestyle throughout childhood and adolescence could prevent expansion of obesity in young adulthood [11].

From previous studies it is obvious that preschool children are more physically active than teenagers and young adults [12,13], but with advancing age, PA decreases significantly in both boys and girls [12,13,14]. This decline in PA is apparent regardless of gender, though preschool boys in general reach higher step counts (SC) than girls [15,16,17].

Furthermore, differences between normal and obese children’s SC on weekdays and weekends are significant already among pre-schoolers [18], which is similar to the pattern observed in schoolchildren [19]. Recent studies on the topic indeed reveal higher pedometer-assessed SC both in European and American preschool boys and girls on weekdays than at weekends [15,16,17,18]. Thus weekends still represent a “critical window” for pre-schoolers to accumulate necessary health-beneficial PA.

Although preschool children spend approximately 5 h per weekday in kindergarten/child-care settings [13,20], parents have an essential influence on their development [21], including adoption of health-enhancing PA [22,23,24,25] and non-excessive ST habits [23,26,27]. The relationships between objectively measured parent-child weekdays-weekend PA [28,29,30,31,32] and parent-child weekdays-weekend sedentary behaviour [33] or ST [34] are well documented in schoolchildren. In contrast, the relationships between parent-child PA [35,36] and ST [26,37] in preschool children are studied rarely. In addition, weekday-weekend differences in the relation between parent-child PA and parent-child ST or even mutual relationships between weekdays-weekends parent-child PA and ST still remain under-researched.

Therefore, the present study aimed to broaden the up-to-date knowledge of the relationship between parents-preschool child PA and ST and answers the research question whether this relationship varies between weekdays and weekend days. The specific objectives were to: (a) describe gender differences in SC and duration of ST in parents and their preschool children on weekdays and at weekends; (b) quantify the association between parents’ SC/ST and children’s SC/ST on weekdays and at weekends.

## 2. Materials and Methods

### 2.1. Ethics

This study was approved by the Ethical Committee of the Faculty of Physical Culture, No. 57/2014. The parents of children and teachers were informed in detail about the study design and parents were required to provide written informed consent, if they and their children wished to participate in this study. Participation was voluntary without any incentives. All participants who completed the research received individual graphical feedback.

### 2.2. Participants and Data Inclusion Criteria

The monitoring of PA in parents and their preschool children was carried out during the April–May and September–October 2015 from two separate subsamples. We invited 296 preschool children (141 girls/daughters) aged 4–7 years from the 10 randomly recruited public kindergartens and their parents (234 mothers and 181 fathers) for participation in the study (Table 1).

The kindergartens involved in our study were selected in order to cover all four Moravian administrative regions of the Czech Republic and include kindergartens both from cities and rural areas. The response rate of participating children and their parents was >75% (223 children; 313 parents). We excluded 29 children and 35 parents from the analyses due to incomplete or invalid data (e.g., missing body weight, height, age; more than a day’s absence in the kindergarten program; insufficient daytime wearing time of pedometer <8 h or daily, and SC of >30,000 or <1000) [38,39]. Participants’ data was included in the study only when the pedometer was worn for at least eight hours a day for at least four weekdays and both weekend days. The anthropometric variables of final dataset of participants are described in Table 1.

### 2.3. Measurements

Parents and their children wore the Yamax Digiwalker SW-200 pedometer (Yamax Corporation, Tokyo, Japan) on the right hip for eight consecutive days. First day of monitoring was excluded from the analyses due to the reactivity effect [40]. Participants were instructed to wear the pedometer for the whole day (except for water-based activities). They started wearing the pedometer in the morning and removed it before going to bed. In addition to pedometer-assessed daily PA, parents and teachers also recorded type and duration of ST behavior to family proxy-reported log book. In accordance with previous studies [8,9,10] ST was chosen as an indicator of sedentary behaviors instead of general inactivity because many health beneficial activities for pre-schoolers are done while sitting or lying down [34,41]. Finally, there is a consensus regarding the definition of excessive “screen time” (≥2 h per day) [42,43]. In contrast, overall time spent being sedentary raises several issues (e.g., problems with valid and reliable data collection, missing consensus on excessive sedentary time, i.e., “how much is too much”). The accuracy of recording the duration of ST activity was fixed at 10 min. Parents/teachers recorded the day time and value of SC shown on the display of the Yamax pedometer in the morning after waking up, start and end of kindergarten/job, and both daily SC and ST in the evening before going to bed [30,31]. Acceptable 14- to 21-day test-retest reliability of seven-day parents’ proxy-reported TV viewing of their 3–8-year-old children has been previously validated (*r_p_* = 0.80, *p* < 0.001) [44]. Parents’ proxy-report of time of duration of seven-day waking sedentary behavior of their preschool children was validated against waking sedentary behavior time assessed by the Actigraph GTIM accelerometer (*r_S_* = 0.24) [44]. The variable of the daily ST represented a sum of sitting and lying while watching TV (DVD, video) and sitting and lying in front of a PC (notebook, tablet, smartphone). Parents’/children’s Body Mass Index (BMI) (kg/m^2^) has been calculated based on self-reported/parent-reported body height (to the nearest 0.5 cm) and weight (to the nearest 0.5 kg). BMI was shown to be a good indicator for identifying children with overweight and obesity [45].

### 2.4. Data Analysis

The data was analyzed using the SPSS v.22 software (IBM SPSS, Inc., Chicago, IL, USA) and STATISTICA v.12 (StatSoft, Prague, Czech Republic). The data was analyzed in total for all kindergartens because the Two Step cluster analysis found no indicator for clustering by kindergarten, child BMI or season in 2015. Descriptive characteristics (M, 95% confidence interval (CI)) for the daily SC and ST variables were calculated separately for daughters, sons, mothers, and fathers on weekdays and weekend days. Daily SC recommendation for preschool children represents a value of 11,500 steps/day [46] and for adults it is a value of 10,000 steps/day [42,47]. An excessive ST for children and adults was defined as two or more hours/day [43,48]. Paired t-test was used for testing the differences between weekday/weekend SC or ST in parents and children, separately for gender. Mann-Whitney U test was used for testing gender-related (parental and child) differences in the daily SC or ST separately for weekdays and weekends. Then, we used the bivariate Pearson correlation to assess the correlations between parents’ and their children’s weekday/weekend SC and ST, respectively, as well as parent’s BMI and children’s ST. Last, we assessed the associations between daily SC and ST of children and their parents, by gender, using linear regression analyses conducted separately for weekdays and weekend days. For graphical presentation of the results of the linear regression analyses, we selected the interval of 2500 steps per day for parents. This interval was used as a cut-off in accordance with the concept of graduated step index for healthy adults [49], which was revised [50] and completed into its final form [51]: (1) <2500 steps/day (“basal activity”); (2) 2500–4999 steps/day (“limited activity”); (3) 5000–7499 steps/day (“low active”); (4) 7500–9999 steps/day (“somewhat active”); (5) 10,000–12,499 steps/day (“active”); and (6) ≥12,500 steps/day (“highly active”). The alpha level of significance was set at the value of 0.05.

## 3. Results

### 3.1. Weekday-Weekend Patterns of Step Counts and Screen Time

Different patterns of behavior were discovered between weekdays and weekends for parents and their children, especially in ST (Table 2). In children and mothers, we found higher daily SC on weekdays than at weekends. However, the weekday-weekend pedometer-determined difference in daily SC was found to be significant (*p* = 0.002) only in mothers (Table 2). Conversely, the same weekday-weekend patterns of daily ST were observed in all family members. Parents and their preschool children spent a significantly longer amount of time (>20 min) in front of TV (DVD, video) or PC and smartphone/tablet monitor at weekends than on weekdays (Table 2). On weekdays, 6.7% of preschool girls and 7.1% of preschool boys spent more than 2 h in front of the screen, while at weekends it was about four times as much in girls (27.0%) and three times as much in boys (21.4%), respectively. Similar weekdays-weekends ST patterns were found also in both the pre-schoolers’ mothers and fathers. More than 2 h of weekdays ST were reported by 17.0% of mothers while at weekends 33.3% of mothers reached more than 2 h of ST. Similarly, a quarter of fathers reported excessive ST on weekdays and, at weekends, it was nearly 50% of them.

### 3.2. Correlations between Parents’ and Children’s Step Counts and Screen Time

Bivariate correlations for daily SC and ST, respectively, stratified by days of week, and children’s and parents’ gender are presented in Table 3. Significant positive associations between both the parent-child SC and parent-child ST were found. Mothers’ and daughters’ SC were more strongly correlated during weekdays, weekends, and the whole week than fathers’ and daughters’ SC.

In addition, except weekend days, mothers also had a more positive relationship with SC of their sons than fathers (Table 3). Fathers’ and daughters’ SC (or ST) was more strongly correlated than father’ and sons’ SC (or ST) on weekdays and also at weekends. In summary, stronger associations between parent-child SC (or ST) are evident at weekends than on weekdays (Table 3).

Positive correlations between the BMI of mothers and ST of their preschool children were found on weekdays (*r* = 0.18; *p* = 0.021), at weekends (*r* = 0.16; *p* = 0.030), and also when assessing the whole week-long measurement (*r* = 0.19; *p* = 0.015). Similar positive correlations were found between father’s BMI and children’s ST during the weekdays (*r* = 0.24; *p* = 0.010) and the whole week (*r* = 0.179; *p* = 0.044).

### 3.3. Quantification of the Association between Parents’ and Children’s Step Counts and Screen Time

**Figure 1:**
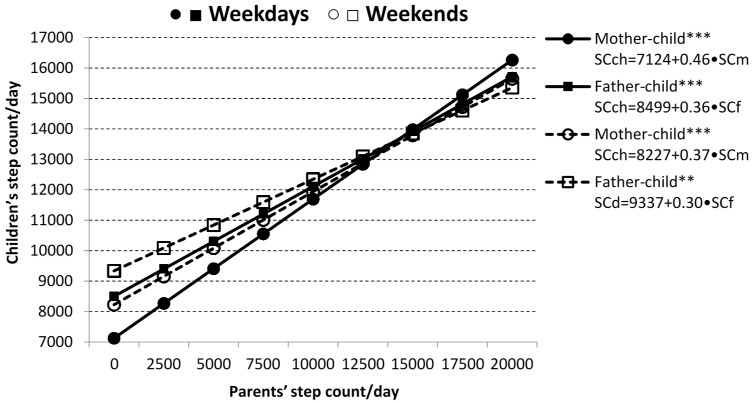
Relationship between parents’ and children’s daily step count (SC). SCch—step count of child; SCf—step count of fathers; SCm—step count of mothers. Statistical significance is expressed as ** *p* < 0.01 and *** *p* < 0.001.

**Figure 2:**
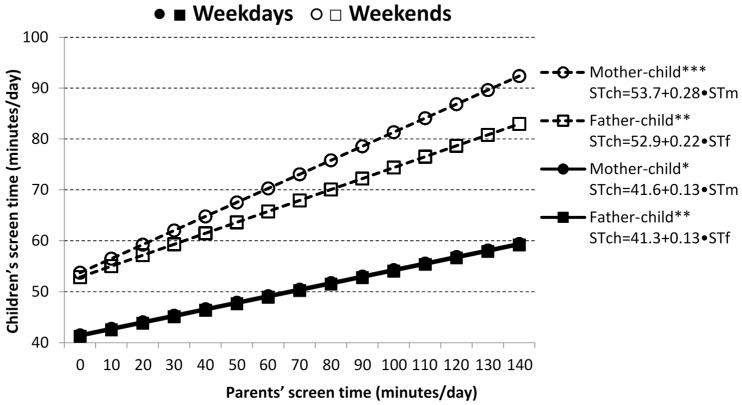
Relationship between parents’ and children’s daily screen time (ST). STch—screen time (min) child; STf—screen time (min) of fathers; STm—screen time (min) of mothers. Statistical significance is expressed as * *p* < 0.05, ** *p* < 0.01 and *** *p* < 0.001.

**Figure 3:**
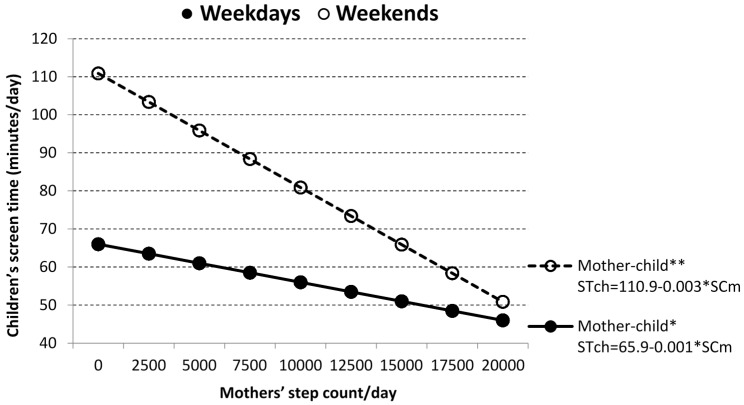
Relationship between children’s daily step count (SC) and mothers’ weekdays (●) and weekends (○) daily screen time (ST). Statistical significance is expressed as * *p* < 0.05 and ** *p* < 0.01.

Results of the linear regression analyses represent the relative association of mothers’/fathers’ energy balance-related behavior (SC or ST) with children’s daily SC and ST, separately for weekdays and weekends (Figure 1, Figure 2 and Figure 3). Mothers’ and fathers’ SC were each significantly associated with children’s SC on weekdays (*p* < 0.001), as well as at the weekends (*p* < 0.001 and *p* < 0.05, respectively). Each 2500 SC increase in mothers’ daily SC on weekdays (at weekends) was linked to a daily SC increase of 1143 (928) in child. A 2500 SC increment in a fathers’ daily SC on weekdays (at weekends) was associated with extra 903 (753) steps per day in child (Figure 1). Results of the linear regression analyses for children’s ST indicated that mothers’ and fathers’ ST was more strongly associated with prolonged ST of their children at weekends than on weekdays (Figure 2).

As expected, each 60-min increase in mothers’/fathers’ ST on weekdays (at weekends) was linked to an increase of 7.6/7.6 (16.8/13.0) proxy-reported minutes of child’s daily ST (Figure 2). Moreover, an increase of 2500 mothers’ daily SC was associated with reduction of 2.5 (7.5) min of child’s weekdays (weekend) ST (Figure 3). Association between fathers’ SC and children’s ST, assessed using linear regression, was also negative as in the case of mother-child pairs, but was significant neither on weekdays, nor at weekends.

## 4. Discussion

The present study aimed at assessing the relationship between parents’ energy balance-related behaviors (SC and ST) and children’s SC and ST, separately on weekdays and at weekends. The results reported here extend previous literature in area of relationship between parent-child weekdays-weekend PA and sedentary behavior. Hitherto, the research has mostly focused on families with school-aged children [28,29,30,31,32] or separately on parent-preschool child ST [26,37] and only a few studies have taken weekday-weekend patterns of pre-schoolers’ PA into account [35,36].

Unlike current European and American studies [15,16,17,18] and in line with previous Czech studies [13,41], pedometer-determined PA was similar on weekdays and at weekends in Czech pre-schoolers. However, as in other studies [15,16,17] we observed higher PA of preschool boys than girls at weekends. The pedometer-determined daily SC of Czech 4–7-year-old preschool children correspond to the daily SC of Canadian boys (12,420 SC/day) and girls (11,438 SC/day) aged 5–7 years [12]. Nevertheless, a proportion of Czech pre-schoolers meeting the recommended target of 11,500 SC/day [46] on weekdays (45.4%) and at weekends (45.9%) was greater than in pre-schoolers from Belgium (40.0%_weekdays_ and 20.5%_weekends_), Bulgaria (29.3%_weekdays_ and 29.2%_weekends_), Greece (26.5%_weekdays_ and 20.3%_weekends_) and comparable to Polish pre-schoolers (43.2%_weekdays_ and 41.8%_weekends_) [17]. At weekends, the percentage of Czech pre-schoolers meeting the 11,500 steps/day recommendation (>37.6%_girls_ and >41.3%_boys_) even surpasses the percentage of those from Germany (31.4%) and Spain (37.0%) [17].

While, in the weekday-weekend patterns of PA of pre-schoolers, country and gender-related differences were uncovered, weekdays-weekends patterns in ST were similar regardless of gender and country of pre-schoolers [17,52] or first-to-second grades schoolchildren [34]. In line with previous studies, Czech preschool boys and girls, as well as their parents, spent much more time in front of TV (DVD, video) or PC and smartphone/tablet monitor at weekends than on weekdays [17,52]. Moreover, a third of participating mothers and almost half of the fathers report more than 2 h of ST a day at weekends. This is similar to the study of Jago et al. [34], which showed that more than 50% of parents of English first-to-second grade children exhibited ≥2 h watching TV [34]. In the Czech preschool children’s parents proxy-reported almost equal ST at weekdays (51.0 min/day) and weekends (78.7 min/day) as parents of the German preschool children (51.8 min/weekday and 79.6 min/weekend), but much lower ST than in Belgian (81.5 min/weekday and 145.2 min/weekend), Bulgarian (107.5 min/weekday and 175.5 min/weekend), Greek (106.0 min/weekday and 163.0 min/weekend), Polish (87.4 min/weekday and 148.4 min/weekend), and Spanish (78.3 min/weekday and 153.2 min/weekend) pre-schoolers [17]. However, a higher daily ST of pre-schoolers from other European countries than daily ST of studied Czech pre-schoolers could be affected by using a less sensitive scale in the applied parent questionnaire (less than 30 min/day; 30 min to <1 h/day; 1–2 h/day; 3–4 h/day; …; 8 h/day; more than 8 h/day [17]), compared with the accuracy of the recording of ST behavior in the Czech parent proxy report (10 min).

In terms of the relationship between parents-pre-schoolers SC and ST, using linear regression we quantified and compared the associations between parents’ SC/ST and children’s SC/ST separately for weekdays and weekends and gender of parents. Although it is difficult to compare the results of methodologically non-identical studies, our results showed a stronger relationship between mother-child PA than father-child PA on weekdays and at weekends, unlike other PA studies of families with schoolchildren [12,28]. However, this finding seems to be logical, because intuitively, care for preschool children (including PA) is closer to mothers than fathers [53]. Thus, because of spending more time with children, mothers might possess more opportunities to influence energy balance-related behaviors of their children.

In addition, for families with preschool children, we did not find gender-specific tendency for PA (closer relationship between father-son than the father-daughter), which was sometimes evident among families with schoolchildren [25,28,54] and adolescents [25,54,55]. Conversely, to the above mentioned studies, we found an even slightly closer relationship between SC and ST of fathers with SC and ST of their daughters than of their sons. However, due to the relatively small sample of study participants this finding cannot be generalized. By monitoring SC and ST in weekdays and weekends, however, it can be argued that more positive associations between parent-preschool child SC (and ST) are evident at weekends than on weekdays as in TV viewing among Greek 3–5-year-old preschool children [52] and English 5–6-year-old school children [34]. It confirms that the behavior of pre-schoolers (including PA and ST) is influenced by many variables besides parental behavior during weekdays (e.g., program in kindergarten), while at weekends the parents apparently might have more chances to stimulate health-enhancing behaviors of their preschool children.

In addition to the previously published studies of PA and sedentary/ST behavior in families with pre-schoolers [26,35,36,37,52] we also investigated the relationship between children’s PA and parents’ ST separately on weekdays and at weekends. Although we observed a negative relationship between fathers’/mothers’ SC and children’s ST, this relationship was significant only in the case of mothers on weekdays (*p* < 0.05) and weekend days (*p* < 0.01).

The findings reported here are therefore comparable to the previous studies in families with pre-schoolers [26,35,36,37,52], but extend the evidence by highlighting how children’s and parents’ SC and ST patterns and the association between parents’ SC/ST and children’s SC/ST varied between weekdays and weekends. Interventions designed to improve PA and nutrition and to promote healthy active living of pre-schoolers are needed to prevent and control childhood obesity [1,56,57,58]. Detection of a weekday-weekend relationship of parent-child PA and ST allows for a deeper description of lifestyle of families with pre-schoolers. Furthermore, a clear quantification of the association of parents’ and children’s SC or ST, respectively might facilitate development of efficient PA programs and interventions. Provided the relationships are causal, the results of the present study suggest that, to promote preschool children’s PA and control their ST, it is appropriate not only to focus on family social support and encouragement [29,32] and apply restrictive rules of children’s ST [26,37], but also to support direct active participation of parents (preferably both) in PA (either in organized programs or in unstructured conditions) on weekdays, as well as at weekends.

## 5. Strengths, Limitations, and Future Research

The major strength of this study is the simultaneous monitoring of indicators of PA (step count) and sedentary behavior (screen time) for both mothers and fathers, which has facilitated the examination of parental associations with children’s behavior. The extension to a younger age group of children could be considered as another strength of the study. Strict criteria for inclusion in the data analysis (data only from children and their parents whose PA and screen time was monitored continuously for at least eight hours a day, on at least four weekdays and both weekend days) can be perceived also as a strength of the study.

As in any paper, the results of this study should be considered with respect to existing limitations. To minimize rejection of voluntary participation in the study information about ethnicity, socio-economic status, marital status, parents’ job, parenting style, and other family factors that might have affected the behavior of both the parents and the pre-schoolers was not investigated. Absence of these potential confounders of children’s behavior is the limit of this study. In addition, the possible influence of the type of residence and quality of neighbourhood on the leisure-time patterns of parent-preschool child PA and sedentary behavior remains unclear.

## 6. Conclusions

This study indicates a clear and quantifiable association between parent-child objectively monitored free-living physical activity and proxy-reported screen time in families with pre-schoolers and how this association varied during weekdays and weekends. Each increase of 2500 steps in mothers’/fathers’ daily step counts at weekdays (weekends) was associated with an extra 1143/903 (928/753) child daily step counts. Each 60 min increase of screen time in mothers’/fathers’ ST at weekdays (weekends) was linked with an extra 7.6/7.6 (16.8/13.0) min of children’s daily min of screen time. An increase of 2500 mothers’ daily step counts was associated with reduction of 2.5 (7.5) min of children’s weekday (weekend) screen time. Although these are limited time-determined data, the findings indicate that efforts spent to increase parents’ physical activity (decrease parents’ screen time) may also increase their preschool children’s physical activity (decrease children’s screen time), and vice versa.

## Figures and Tables

**Table 1:** Descriptive characteristics (number; %; means and standard deviations) of participants.

Variables	Mothers	Fathers	Daughters	Sons
Number (%) of addressed respondents	234 (100%)	181 (100%)	141 (100%)	155 (100%)
Number (%) of participants with informed consent	183 (78.2%)	130 (71.8%)	103 (73.1%)	120 (77.4%)
Number (%) of participants who returned pedometers and met inclusion criteria	166 (70.9%)	112 (61.9%)	88 (62.4%)	106 (68.4%)
Anthropometric variables of final dataset	(*n* = 166)	(*n* = 112)	(*n* = 88)	(*n* = 106)
Age (years)	36.1 ± 4.3	38.7 ± 4.9	5.6 ± 0.8	5.6 ± 0.9
Body height (cm)	167.7 ± 6.2	180.4 ± 6.8	116.3 ± 10.5	117.5 ± 7.6
Body weight (kg)	67.9 ± 11.7	84.9 ± 12.2	20.5 ± 3.9	21.4 ± 3.8
Body Mass Index (kg/m^2^)	24.2 ± 4.1	26.0 ± 3.3	15.2 ± 2.4	15.4 ± 1.8

Notes: n—number of participants; %—percentages.

**Table 2:** Weekday-weekend patterns of daily step counts and screen time (mean and 95% CI) in parents and their preschool children.

Family Members	Step Counts (Number per Day)			Screen Time (Minutes per Day)		
	Weekdays M (95% CI)	Weekends M (95% CI)	*p*-Level	Weekdays M (95% CI)	Weekends M (95% CI)	*p*-Level
*Parents*						
Mothers (*n* = 166)	10,588 (9928–11248)	9624 (8935–10,313)	0.002	69.64 (53.27–86.01)	93.64 (73.63–113.65)	0.001
Fathers (*n* = 112)	10,283 (9573–10993)	10,376 (9628–11,124)	0.794	89.98 (71.38–108.53)	112.02 (94.65–129.39)	0.006
*Children*						
Daughters (*n* = 88)	11,456 (10,705–12,207)	10,749 (9816–11,683)	0.085	45.66 (37.47–53.85)	43.51 *** (22.26–61.87)	<0.001
Sons (*n* = 106)	12,359 (11,531–13,187)	12,089 * (11,227–12,951)	0.624	55.75 (46.72–64.78)	78.77 (63.70–93.84)	<0.001

Notes: n—number of participants; M—mean; 95% CI—confidence intervals; *p*-level—level of statistical significance of comparison of weekdays-weekend step counts (screen time) by paired *t*-test; statistical significance of gender-related (parental/child) differences in step counts (screen time) on weekdays/at weekends is expressed as * *p* < 0.05 and *** *p* < 0.001.

**Table 3:** Bivariate (Pearson) correlations with 95% confidence intervals (95% CI) between parents’ and children’s daily step counts and screen time.

Variable	Step Counts (Number per Day)			Screen Time (Minutes per Day)		
	Daughters	Sons	All	Daughters	Sons	All
*Weekdays*						
Mothers	0.47 *** (0.27–0.63)	0.37 *** (0.17–0.53)	0.39 *** (0.25–0.51)	0.15	0.31 ** (0.08–0.51)	0.21 ** (0.05–0.37)
Fathers	0.34 ** (0.06–0.56)	0.24 (−0.02–0.46)	0.29 ** (0.11–0.45)	0.49 ** (0.22–0.69)	0.15	0.28 ** (0.08–0.46)
*Weekends*						
Mothers	0.69 *** (0.55–0.79)	0.25 ** (0.04–0.43)	0.46 *** (0.33–0.57)	0.27 * (0.03–0.48)	0.43 *** (0.22–0.61)	0.34 *** (0.185–0.48)
Fathers	0.46 *** (0.20–0.65)	0.29 * (0.04–0.50)	0.36 *** (0.19–0.51)	0.57 *** (0.32–0.75)	0.17	0.31 ** (0.11–0.48)
*Week*						
Mother	0.58 *** (0.41–0.71)	0.35 *** (0.15–0.52)	0.44 *** (0.31–0.56)	0.26 * (0.02–0.48)	0.41 *** (0.19–0.59)	0.33 *** (0.16–0.47)
Father	0.39 ** (0.12–0.60)	0.23 (−0.02–0.45)	0.31 *** (0.13–0.47)	0.63 *** (0.40–0.79)	0.18	0.38 *** (0.18–0.54)

Notes: Statistical significance is expressed as * *p* < 0.05, ** *p* < 0.01 and *** *p* < 0.001.
